# Supplementary material for: Colored Shade Nets Can Relieve Abnormal Fruit Softening and Premature Leaf Senescence of “Jumeigui” Grapes during Ripening under Greenhouse Conditions
Source: Plants (Basel). 2022 May 1;11(9):1227. doi: 10.3390/plants11091227 (PMC9102565; doi:10.3390/plants11091227)
Supplement: Supplementary file 1 [file plants-11-01227-s001.zip › plants-1674270-supplementary.pdf]

plants

Article

Colored Shade Nets Can Relieve Abnormal Fruit Softening and Premature Leaf Senescence of "Jumeigui" Grapes during Ripening under Greenhouse Conditions

Qian Zha [1,2], Xiangjing Yin [1,2], Xiaojun Xi [1,2,*] and Aili Jiang [1,2,*]

1 Research Institute of Forestry and Pomology, Shanghai Academy of Agricultural Sciences, Shanghai 201403, China; zhaqian@saas.sh.cn (Q.Z.); yinxiangjing@saas.sh.cn (X.Y.).
2 Shanghai Key Labs of the Protected Horticultural Technology, Shanghai Academy of Agricultural Sciences, Shanghai 201403, China.
* Correspondence: xxj220401@126.com (X.X.); putaojal@163.com (A.J.)

Supplementary Material

Table S1. Definitions of chlorophyll *a* fluorescence parameters.

Parameter	Definition
$F_v/F_m = \Phi_{Po}$	Maximum photochemical efficiency
Ψ_{Eo}	The ratio of excitons captured by the reaction center and used to promote electron transfer to other electron acceptors in the electron transport chain exceeding Q_A to promote Q_A reduction excitons (t = 0).
Φ_{Eo}	Quantum yield for electron transfer (t = 0).
δ_{Ro}	Probability of electrons passing from the intersystem electron transfer body to the electron acceptor on the acceptor side of the PSI.
W_k	Ratio of K-phase variable fluorescence to J-phase variable fluorescence.
Mo	Approximate initial slope of transient fluorescence, V = f(t) (per ms). This parameter reflects the closing rate of PSII active reaction centers (RCs).
PI_{ABS}	Performance index based on absorption of light energy

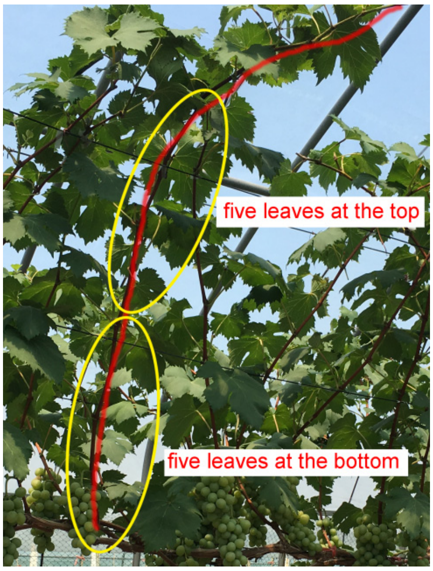

Figure S1. Distribution of the five leaves at the top and five leaves at the bottom on the fruiting branches of grapes.

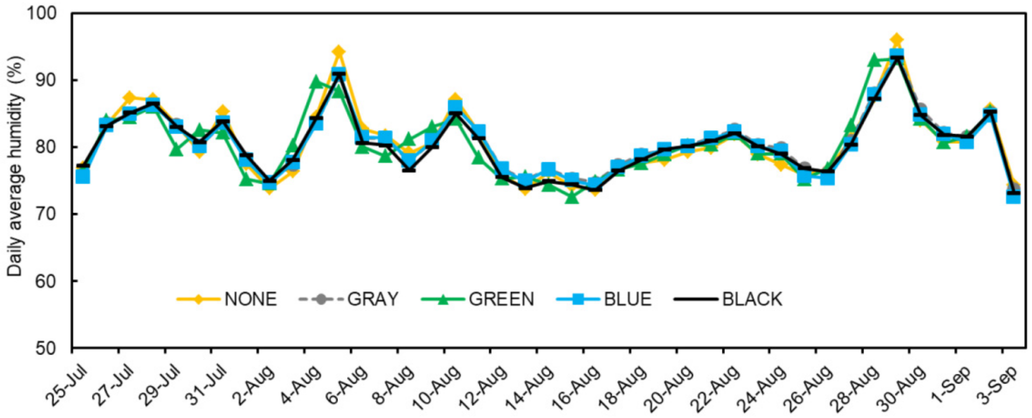

Figure S2. Daily average humidity trend in the facility greenhouse under the colored shading materials.

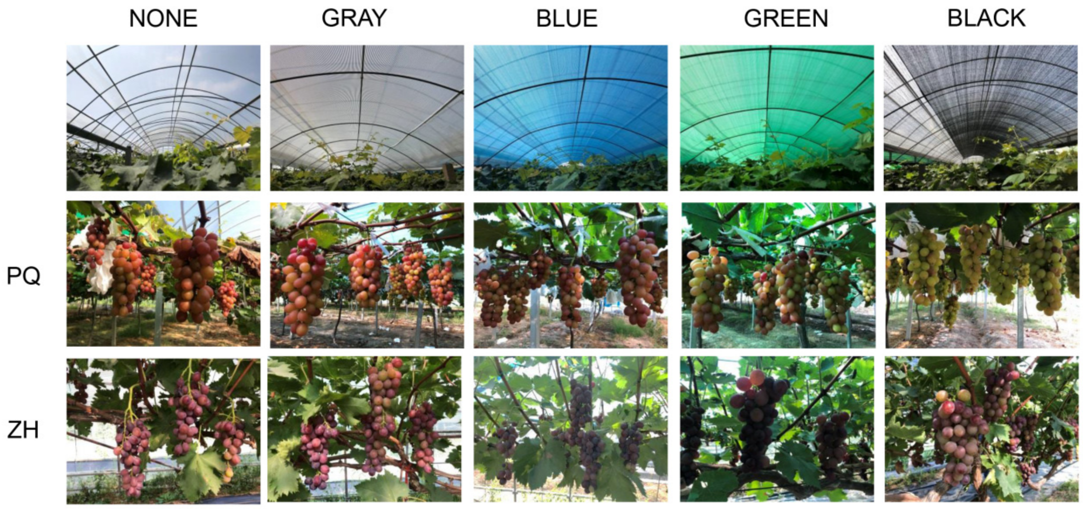

Figure S3. Field performance of grape berries under different treatments.

NONE: no shading; GRAY, BLUE, GREEN, and BLACK: shade net colors. PQ and ZH: experimental vineyard sites at the Shanghai Pingqi Grape Planting Professional Cooperative (30°11′N, 121°25′E), and at Zhuanghang, the site at the Shanghai Academy of Agricultural Sciences, Shanghai, China (30°51′N, 121°13′E), respectively.
